# Long-Term Traditional Chinese Medicine Combined with NA Antiviral Therapy on Cirrhosis Incidence in Chronic Hepatitis B Patients in the Real-World Setting: A Retrospective Study

**DOI:** 10.1155/2020/3826857

**Published:** 2020-08-03

**Authors:** Zhi-Jun Hou, Jing-Hao Zhang, Xin Zhang, Qi-Hua Ling, Chao Zheng, Xiao-Jun Zhu, Zhen-Hua Zhou, Man Li, Xiao-Ni Kong, Xue-Hua Sun, Yue-Qiu Gao, Jian-Jie Chen

**Affiliations:** ^1^Department of Hepatopathy, Shuguang Hospital, Affiliated to Shanghai University of Traditional Chinese Medicine, Shanghai 201203, China; ^2^Laboratory of Cellular Immunity, Shanghai Key Laboratory of Traditional Chinese Medicine, Affiliated to Shanghai University of Traditional Chinese Medicine, Shanghai 201203, China; ^3^Shanghai Traditional Chinese Medicine Clinical Center of Hepatopathy, Affiliated to Shanghai University of Traditional Chinese Medicine, Shanghai 201203, China; ^4^Central Laboratory, Shuguang Hospital, Affiliated to Shanghai University of Traditional Chinese Medicine, Shanghai 201203, China

## Abstract

**Objective:**

To evaluate the impact of long-term Traditional Chinese Medicine (TCM) syndrome differentiation combined with antiviral therapy with Nucleos (t) ide analogues (NAs) on the incidence of cirrhosis in patients with chronic hepatitis B.

**Methods:**

This retrospective cohort study included 521 patients with chronic hepatitis B who underwent a treatment course of ≥3 years from 1998–2019. Of the 521 patients, 261 were defined as TCM users while 260 were TCM nonusers (control group). All the enrolled subjects were followed up until February 2019 to measure the incidence and hazard ratio (HR) of cirrhosis, and the Cox proportional hazards regression model was used to analyze the independent factors affecting the occurrence of cirrhosis.

**Results:**

The cumulative incidence of TCM users and nonusers was 6.9% and 13.5%, respectively (*P*=0.013). Results of the Kaplan–Meier analysis demonstrated that TCM users had a significantly lower cumulative incidence of cirrhosis than TCM nonusers (*P*=0.011), and TCM users had a significantly lower liver cirrhosis risk than TCM nonusers (adjusted HR = 0.416, 95% CI, 0.231–0.749). The histological evaluation revealed improved fibrosis in 45.0% of TCM users and 11.1% of TCM nonusers (*P*=0.033). The analysation of the prescriptions including total 119 single Chinese herbs medicinal demonstrated that “replenish qi and fortify the spleen,” “clear heat and dispel dampness,” and “soothe the liver and regulate qi” are the main treatment methods of TCM for CHB.

**Conclusions:**

Our study demonstrated that long-term TCM use may attenuate liver cirrhosis risk in patients with chronic hepatitis B (CHB).

## 1. Introduction

Hepatitis B virus (HBV) infection is a public health concern worldwide, and persistent HBV infection can cause chronic hepatitis B (CHB), which is a serious threat to human health, leading to a huge social burden. According to the latest data in 2018, there were approximately 292 million hepatitis B carriers worldwide in 2016, and the rate of hepatitis B surface antigen positivity in China was 6.1%, indicating that there were approximately 81 million people with HBV infection [1]. HBV-related cirrhosis is a major clinical outcome of chronic HBV infection. According to the World Health Organization (WHO) estimates, approximately 30% of patients worldwide who died of cirrhosis in 2002 were HBV-associated cirrhosis [2]. The annual incidence of cirrhosis in patients with CHB is 2–10% [3]. The 5-year cumulative cirrhosis rate of patients with chronic HBV infection is 8–20%.

Several guidelines for hepatitis B prevention emphasize that the long-term treatment goal of CHB is to continuously inhibit and eliminate HBV DNA and to prevent and delay complications such as cirrhosis and HCC [4–6]. The value and status of antiviral treatment for CHB has been widely recognized. Nucleos (t) ide analogues (NAs) and poly-pegylated interferon (PEG-IFN) can inhibit HBV replication, thereby reducing the pathological damage to the liver. However, the persistence of covalently closed circular DNA (cccDNA) in hepatocytes is the main cause of prolonged relapse and easy recurrence of CHB after treatment is discontinued [7]. Numerous clinical trials have demonstrated that although long-term NA therapy has been shown to improve long-term clinical outcomes, long-term antiviral therapy alone does not completely prevent the occurrence of cirrhosis and HCC and face the challenge of patients' poor compliance.

Traditional Chinese medicine (TCM) is based on syndrome differentiation, and it emphasizes an individualized treatment and holistic view. According to the diagnosis and treatment guidelines of TCM for CHB, the main mechanism and syndrome type of CHB are liver-gallbladder dampness-heat, liver depression, and spleen deficiency [8, 9]. TCM can play complementary roles in antiviral treatment, facilitate disease recovery, and delay its progress. Thus far, there are no specific antifibrotic chemical or biological drugs that can be used in clinical practice, and Chinese medicine has clear advantages in this field [10]. The combination of traditional Chinese and Western medicine has gradually become the main way to treat CHB in China. This approach can delay the progress of CHB and improve clinical prognosis. However, no long-term follow-up observations have been performed to verify the efficacy of this approach.

Thus, the present study aimed to determine the impact of long-term TCM syndrome differentiation combined with NA antiviral therapy on the incidence of cirrhosis in CHB patients.

## 2. Methods

### 2.1. Study Subjects

This retrospective cohort study was generated from real-world practice using the registration and inpatient and outpatient datasets from 1998–2019 from the Shanghai Shuguang Hospital and the Shanghai Pudong New District Infectious Diseases Hospital, China. Data obtained for analysis included data from electronic medical records (EMR), laboratory information system (LIS), and picture archiving and communication systems (PACS), as well as the detailed procedures, medication, and TCM prescriptions from paper-based medical records.

**Figure 1:**
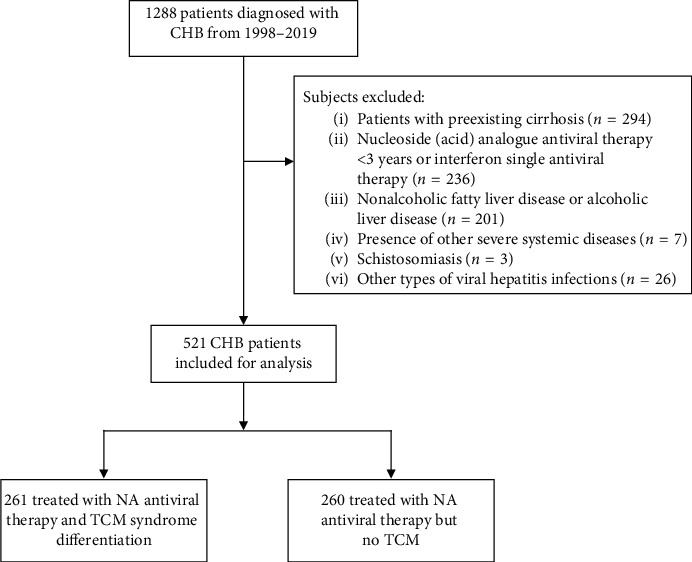
Flowchart of patient recruitment for the current study.

This cohort study screened 1288 patients with CHB between 18 and 65 years of age. Of these, 767 patients were excluded from the study based on the following exclusion criteria: presence of cirrhosis, nucleoside (acid) antiviral therapy for <3 years or single antiviral therapy with interferon; concomitant nonalcoholic fatty liver disease or alcoholic liver disease, presence of other severe systemic diseases, presence of schistosomiasis, and other types of viral hepatitis infection. The remaining 521 patients were diagnosed with CHB and had been receiving continuous treatment with NA for more than three years either with or without TCM; these patients were deemed eligible and were included in the data analysis.  Figure 1 presents the flowchart of patient recruitment for this study.

According to the different degrees of exposure to TCM syndrome differentiation in clinical practice, patients were divided into two cohorts based on whether they received a combination of Chinese and Western medicine or whether they received Western medicine alone. Patients who received Chinese herbal medicine for HBV infection-related diseases for more than 3 months per year for ≥3 years were designated as TCM users. Patients who were exposed to TCM for less than 3 months per year regardless of the total treatment course were designated as TCM nonusers.

### 2.2. Efficacy Measures

The primary outcome was the cumulative incidence of cirrhosis in patients with CHB. Cirrhosis was mainly defined by ultrasonography, computed tomography (CT), or magnetic resonance imaging (MRI) of the liver. The second outcome was histological response during follow-up, and a positive response was defined as an improvement in necroinflammation by at least one grade in the Scheuer system for necroinflammation or improvement in fibrosis by at least one grade according to the Scheuer fibrosis staging classification.

### 2.3. Statistical Analyses

Statistical data were described using means ± standard deviation, medians, or percentages. Differences in the baseline characteristics between the two groups were assessed by Student's *t*-test or chi-square test. The cumulative incidence of cirrhosis was determined using the Kaplan–Meier algorithm, and the log-rank test was used to evaluate the difference between the two groups. The independent factors affecting the incidence of cirrhosis were analyzed by multivariate analysis using the LR forward method of the Cox proportional hazards regression model. Data were statistically analyzed using SPSS v21.0 (IBM, Armonk, NY, USA). A *P* value of <0.05 (two-sided) was considered statistically significant.

## 3. Results

Table 1 describes the baseline characteristics of study patients. There were no significant differences between the two groups. The follow-up duration of patients who received a combination of traditional Chinese and Western medicine ranged from 3–21 years with a median follow-up of 8 years; that of patients who received Western medicine alone was 3–19 years with a median follow-up of 8 years (*P* > 0.05). The antiviral treatment duration with antiviral therapy was 3.1–20.6 years (median, 6.6 years) in the cohort that received Chinese and Western medicine and 3–18 years (median, 7 years) in the cohort that received Western medicine alone (*P* > 0.05).

**Figure 2:**
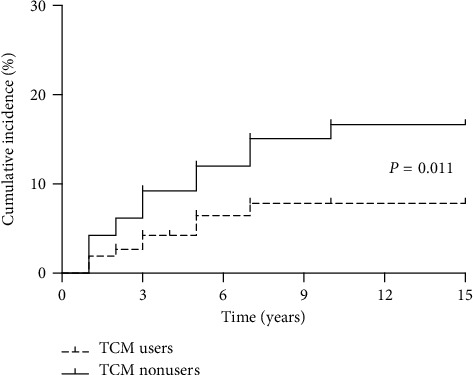
Cumulative incidence of cirrhosis in CHB patients with and without TCM syndrome differentiation. The incidence of liver cirrhosis was significantly lower (*P*=0.011) in TCM users (*n* = 261) than in TCM nonusers (*n* = 260).

**Figure 3:**
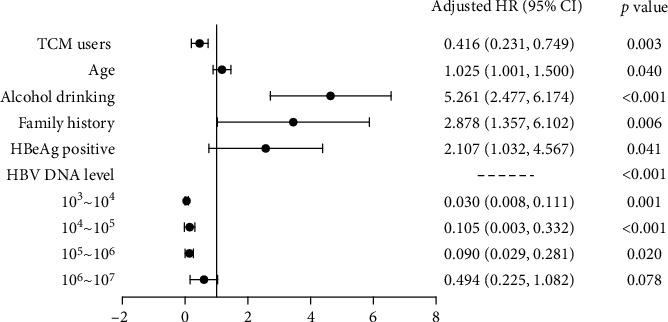
Multivariable predictor of liver cirrhosis. Results of the multivariable predictor of liver cirrhosis according to TCM utilization status along with the baseline characteristics, as shown in Tables 1 and 2.

A total of 53 liver cirrhosis events (18 in TCM users and 35 in TCM nonusers) occurred during the follow-up period. The cumulative incidence of cirrhosis was 6.9% in TCM users versus 13.5% in TCM nonusers (chi-square = 6.143, *P*=0.013). Kaplan–Meier analysis demonstrated that TCM users had a lower cumulative incidence of cirrhosis than TCM nonusers (chi-square test = 6.534, *P*=0.011; Figure 2).  Table 2 illustrates the incidence of cirrhosis among patients with different baseline characteristics.  Figure 3 illustrates the results of multivariable predictor of liver cirrhosis incidence with respect to the TCM utilization status along with the baseline characteristics in Tables 1 and 2. TCM users had a significantly lower liver cirrhosis risk than TCM nonusers (adjusted HR = 0.416, 95% CI, 0.231–0.749).

**Figure 4:**
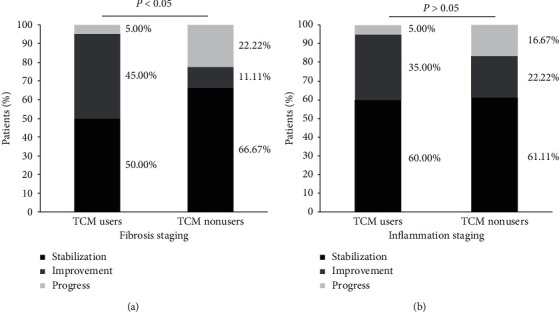
Histological response of CHB patients with and without TCM use. A total of 38 patients had paired liver biopsy (20 and 18 in TCM users and nonusers, respectively). Histological evaluation showed that 45.0% of TCM users and 11.1% of TCM nonusers achieved improved fibrosis (*P*=0.033) (a) and 35.0% of TCM users and 22.2% of TCM nonusers achieved improvement in necroinflammation (*P*=0.627) (b).

A total of 144 patients underwent liver biopsy at baseline (71 and 74 in the TCM user and nonuser groups, respectively). During the follow-up, 38 patients underwent paired liver biopsies (20 and 18 in the TCM user and nonuser groups, respectively). The results of the histological evaluation (Figure 4) revealed that 35.0% of TCM users and 22.2% of TCM nonusers achieved improvements in necroinflammation (*P*=0.627) and 45.0% of TCM users and 11.1% of TCM nonusers achieved improvements in fibrosis symptoms (*P*=0.033).

A total of 119 single Chinese herbs were collected in all the TCM prescriptions for CHB patients. The names and major functions of the mostly used single Chinese herbs are shown in Table 3, which were mainly heat-clearing medicinal, dampness-resolving medicinal, spleen-fortifying medicinal, and qi-regulating medicinal. It was demonstrated that “replenish qi and fortify the spleen,” “clear heat and dispel dampness,” and “soothe the liver and regulate qi” are the main treatment methods of TCM for CHB.

## 4. Discussion

CHB is an immune-mediated disease, and the interaction of the HBV with innate and acquired immune responses determines the clinical outcome of HBV infection. There are three obstacles for curing CHB: the persistence of the virus cccDNA, dysfunction of the immune response to HBV, and pathological changes in liver tissue (inflammation, necrosis, fibrosis, and cirrhosis). The development of HBV infection from chronic hepatitis to cirrhosis and even to liver cancer is a multistage pathogenic process and is affected by many factors, including virus (such as the HBV genotype, viral mutation, viral load, and viral proteins levels), host (such as age, gender, genetic polymorphism, presence or absence of other viral infections, and intestinal microecology), and other exogenous factors, and also whether or not patients receive antiviral therapy in a timely and standardized manner [11]. The HBV DNA load, serum HBsAg level, HBeAg statue, and ALT levels have been shown to be closely related to the clinical outcome of CHB [12].

Antiviral therapy is the key to preventing disease progression and improving the prognosis of patients with CHB. A number of studies have shown that long-term antiviral therapy can substantially improve the degree of liver fibrosis in patients with CHB. In a long-term follow-up study (mean follow-up duration 89.9 months) of CHB patients, the incidence of cirrhosis and HCC significantly reduced in CHB patients who received lamivudine compared with patients who did not receive antiviral therapy [13]. It was reported that, among the CHB patients treated with entecavir for 5 years, 88% showed improvement in liver fibrosis and 40% achieved cirrhosis reversal [14]. A 5-year open-label follow-up study of tenofovir in patients with CHB revealed that long-term inhibition of HBV can improve histology and reverse liver fibrosis and cirrhosis [15]. However, long-term use of NA antiviral therapy has gradually exposed problems such as drug resistance, viral relapse, and adverse reactions. Some studies also showed that even with active antiviral therapy, patients have varying degrees of liver histological lesions [16–19], and the long-term prognosis of CHB patients with nonantiviral indications is not optimistic.

TCM has a long history of treating CHB. TCM has over time formed a unique diagnosis and is associated with treatment advantages in long-term clinical practice. TCM plays an important role in regulating immune function, improving liver function, exerting antifibrosis and antiviral effects, and eliminating the clinical signs and symptoms of patients. A systematic review of TCM with NA therapy for treating CHB revealed that it is better than NA therapy alone in terms of the improving liver function, HBeAg seroconversion rate, HBV DNA negative conversion rate, and the incidence of YMDD mutation [20]. According to TCM theory, hepatitis B-related cirrhosis locations include the liver, spleen, and kidney, and the pathological products of hepatitis B-related cirrhosis include blood stasis, water, stagnation of qi, and dampness of phlegm. The basic pathogenesis is qi deficiency and blood stasis and phlegm blocking. Studies have shown that the application of TCM such as Fuzheng Huayu, Fufang Biejia Ruangan pill, Dahuang Zhechong, Anluo Huaxian wan, and Biejia Jianwan can prevent liver fibrosis and block the link between CHB and liver cirrhosis [21–25]. Currently, relevant clinical studies on CHB treatment with Chinese medicine or Chinese medicine integrated with Western medicine have generally covered a short treatment course of 6 or 12 months. No reports have investigated the long-term effectiveness and safety of TCM in the treatment of CHB.

Here, our study attempted to further reveal and objectively evaluate the role of TCM in delaying the progression of CHB disease by observing the occurrence of cirrhosis in CHB patients who undergo long-term TCM syndrome differentiation combined with antiviral therapy. This study demonstrated that the cumulative incidence of cirrhosis was significantly lower among TCM users than among TCM nonusers (6.9% *versus* 13.5%, *P*=0.013), and TCM users had a significantly lower liver cirrhosis risk than TCM nonusers (adjusted HR = 0.416, 95%CI 0.231 to 0.749). With regard to the histological response, there was no significant difference in improvement of necroinflammation between the two cohorts (35.0% *vs*. 22.2%, *P*=0.627), but 45.0% of TCM users achieved improved fibrosis, which was significantly higher than the percentage of TCM nonusers who achieved improved fibrosis (11.1%; *P*=0.033). Although, it is difficult to determine an effective commonly used TCM description, but through analyzing the use of Chinese herbs in the prescription, it was demonstrated that “replenish qi and fortify the spleen,” “clear heat and dispel dampness,” and “soothe the liver and regulate qi” are the main methods of treatment for CHB, which were consistent with the guidelines of TCM for CHB [9].

This study has several limitations. First, it is not a randomized and controlled trial; thus, there may be potential bias due to unrecognized confounding factors such as household income, educational background, and occupation. As these factors are more likely to affect TCM use, they must be included in further studies. Second, given the deficiencies of a retrospective study, patient information may be incomplete and there could be biases in data collection and on follow-up; thus, we used registration and inpatient and outpatient claims datasets, as well as paper-based medical records to reduce this bias as much as possible. Third, this study did not consider over-the-counter TCM use for herbal medicines and traditional Chinese patent medicine; thus, the prevalence of TCM may be underestimated. Fourth, syndrome differentiation according to TCM is based primarily on the physicians' individual experiences: one physician may prescribe different prescriptions at each visit, and one patient could visit different physicians during the long-term follow-up period. This could result in using very small proportion of each prescription, and it is difficult to even determine a common prescription that is effective, let alone identifying the specific effective components in each herb. Finally, due to the limited access to data, our study only operated in the Shanghai Shuguang Hospital (Grade III Level A) and the Shanghai Pudong New District Infectious Diseases Hospital (Grade II Level A). Further multicenter studies need to be carried out to verify the effect of TCM.

## 5. Conclusions

In conclusion, this retrospective study demonstrated that the long-term use of syndrome differentiation according to TCM could attenuate the liver cirrhosis risk in patients with CHB. The findings of this study should be further validated by randomized controlled trails.

## Figures and Tables

**Table 1:** Baseline characteristics of two groups.

Characteristics	TCM users (*n* = 261)	TCM nonusers (*n* = 260)	*P* value
Age (years)			0.307
<40	164 (62.8)	152 (58.5)	
≥40	97 (37.2)	108 (41.9)	

Gender (*n*, %)			0.670
Male	167 (64.0)	171 (65.8)	
Female	94 (36.0)	89 (34.2)	

Duration of illness (*n*, %)			0.757
0∼5	62 (23.8)	70 (26.9)	
5∼10	55 (52.1)	48 (18.5)	
10∼15	59 (22.6)	62 (23.8)	
15∼	85 (32.6)	80 (30.8)	

Smoking history (*n*, %)			0.506
Yes	33 (12.6)	28 (10.8)	
No	228 (87.4)	232 (89.2)	

Drinking history (*n*, %)			0.487
Yes	45 (17.2)	39 (15.0)	
No	216 (82.8)	221 (85.0)	

Family history of CHB-related diseases (*n*, %)			0.549
Yes	96 (36.8)	101 (39.8)	
No	165 (63.2)	159 (61.2)	

HBeAg			0.749
Positive	153 (58.6)	156 (60.0)	
Negative	108 (41.4)	104 (40.0)	

HBV DNA level (copies/mL)			0.644
10^3^∼10^4^	39 (14.9)	35 (13.5)	
10^4^∼10^5^	31 (11.9)	29 (11.2)	
10^5^∼10^6^	34 (13.0)	46 (17.7)	
10^6^∼10^7^	45 (17.2)	49 (18.8)	
10^7^	112 (42.9)	101 (38.8)	

ALT (IU/ml ULN)			0.714
1∼2	48 (18.4)	54 (20.8)	
2∼3	137 (52.5)	129 (49.6)	
3∼	76 (29.1)	77 (29.6)	

**Table 2:** Incidence of cirrhosis for CHB patients with different baseline characteristics.

Characteristics	Cirrhosis (*n* = 53)	Noncirrhosis (*n* = 468)	*P* value
Age (years)			0.965
<40	32 (10.1)	284 (89.9)	
≥40	21 (10.2)	184 (89.8.9)	

Gender (*n*, %)			0.021
Male	42 (12.4)	296 (65.8)	
Female	11 (6.0)	172 (94.0)	

Duration of illness (*n*, %)			0.158
0∼5	5 (3.8)	127 (96.2)	
5∼10	9 (8.7)	94 (91.3)	
10∼15	14 (11.6)	107 (88.4)	
15∼	25 (15.2)	140 (84.8)	

Smoking history (*n*, %)			0.419
Yes	8 (12.6)	53 (10.8)	
No	45 (87.4)	415 (89.2)	

Drinking history (*n*, %)			0.032
Yes	14 (16.7)	70 (83.3)	
No	39 (82.8)	398 (91.1)	

Family history of CHB-related diseases (*n*, %)			0.016
Yes	27 (13.7)	170 (86.3)	
No	26 (8.0)	298 (92.0)	

HBeAg			0.027
Positive	39 (12.5)	270 (87.5)	
Negative	14 (6.6)	198 (93.4)	

HBV DNA level (copies/mL)			0.022
10^3^∼10^4^	3 (4.1)	71 (95.9)	
10^4^∼10^5^	4 (3.3)	56 (96.7)	
10^5^∼10^6^	6 (7.5)	74 (92.5)	
10^6^∼10^7^	11 (11.7)	83 (88.3)	
10^7^	29 (14.6)	184 (85.4)	

ALT (IU/ml ULN)			0.473
1∼2	8 (7.8)	94 (92.2)	
2∼3	26 (9.8)	240 (90.2)	
3∼	19 (12.4)	134 (87.6)	

**Table 3:** The names and major functions of single Chinese herb medicinal mostly used in TCM prescriptions for CHB patients.

Chinese herb medicinal	Major functions	No. of users	%
*Atractylodes macrocephala Koidz.* (Bai Shu)	Fortify the spleen and replenish qi, dry dampness, and induce diuresis	180	68.97
*Atractylodes lancea* (*Thunb.*) *DC.* (Cang Shu)	Dry dampness and fortify the spleen, dispel wind, and disperse cold	153	58.62
*Corydalis yanhusuo* W. T. Wang ex Z. Y. Su et C.Y. (Yan Hu Suo)	Active blood and resolve stasis and move qi to relieve pain	129	49.43
*Artemisia capillaris* Thunb. (Yin Chen)	Clear heat and drain dampness, drain bile, and anti-icteric	111	42.53
*Poria cocos* (*Schw.*) *Wolf* (Fu Ling)	Induce diuresis to dry dampness, fortify spleen, and tranquilize heart	94	36.02
*Gardenia jasminoides* Ellis (Zhi Zi)	Purge fire and except vexed, clear heat and drain dampness, and cool blood and detoxify	82	31.42
*Serissa japonica* (Thunb.) Thunb. (Liu Yue Xue)	Disperse wind and release the exterior, clear heat and drain dampness, stimulate the circulation of the blood, and cause the muscles and joints to relax	79	30.72
*Cleistocactus sepium* (Wu Zei Gu)	Stop bleeding with astringency, inhibit acidity to relieve pain, drain dampness, and disperse abscesses	72	27.59
*Pinellia ternata* (Ban Xia)	Dry dampness to resolve phlegm, direct qi downward to relieve emesis, and disperse stuffiness and nodules	67	25.67
*Scutellaria barbata* D. Don (Ban Zhi Lian)	Clear heat and detoxify and resolve stasis to induce diuresis	64	24.52
Spreading Hedyotis Herb (She She Cao)	Clear heat and drain dampness and detoxify	57	21.84
*Citrus reticulata Blanco* (Qing Pi)	Soothe the liver and regulate qi and disperse accumulation and stagnation	55	21.07
*Citrus reticulata Blanco* (Chen Pi)	Regulate qi and fortify the spleen and dry dampness to resolve phlegm	45	17.24
*Lysimachia christinae* Hance (Jin Qian Cao)	Drain dampness and anti-icteric and induce diuresis to relieve strangury	45	17.24
*Bupleurum chinense* (Chai Hu)	Harmonize and release the exterior and interior, soothe the liver to relieve depression, raise yang and lift prolapsed organs, and interrupt malaria	44	16.86
*Glycyrrhiza uralensis* Fisch (Gan Cao)	Fortify the spleen and replenish qi, clear heat and detoxify, dispel phlegm to suppress cough, relieve spasm and pain, and moderate herbs	43	16.48
*Setaria italica* (*L.*) *Beauv.* (Gu Ya)	Promote digestion, invigorate the stomach, and increase the appetite	41	15.71
*Plantago depressa* Willd. (Che Qian Cao)	Clear heat, induce diuresis, dispel phlegm, and cool blood and detoxify	40	15.33

## Data Availability

The datasets used and analyzed during this study are available from the corresponding authors upon reasonable request.

## Abbreviations

ALT: Alanine aminotransferase
CHB: Chronic hepatitis B
CT: Computed tomography
cccDNA: Covalently closed circular DNA
EMR: Electronic medical record
HBV: Hepatitis B virus
HBeAg: Hepatitis B e antigen
HBsAg: Hepatitis B surface antigen
HCC: Hepatocellular carcinoma
HR: Hazard ratios
LIS: Laboratory information system
MRI: Magnetic resonance imaging
NAs: Nucleos (t) ide analogues
PACS: Picture archiving and communication systems
PEG-IFN: Poly-pegylated interferon
TCM: Traditional Chinese medicine
WHO: World Health Organization
95%CI: 95% confidence interval.
